# Spinopelvic dissociation: extended definition, physical examination, classification, and therapy

**DOI:** 10.1186/s13018-023-03523-z

**Published:** 2023-01-19

**Authors:** Bin Shi, Ye Peng, Gongzi Zhang, Shuwei Zhang, Yang Luo, Faqin Lv, Ying Hu, Lihai Zhang

**Affiliations:** 1 Department of Orthopedics, Chinese PLA General Hospital, No. 28 Fuxing Road, Beijing, 100853 People’s Republic of China; 2 Department of Rehabilitation Medicine, Chinese PLA General Hospital, Beijing, 100853 People’s Republic of China; 3 Department of Ultrasonography, The No. 3 Medical Center of Chinese PLA General Hospital, Beijing, 100039 People’s Republic of China; 4 Shenzhen Institute of Advanced Technology, Chinese Academy of Sciences, Shenzhen, 518055 People’s Republic of China

**Keywords:** Spinopelvic dissociation, Special physical examination, 301SPD classification, Penetration screw, Spondylopelvic fixation

## Abstract

**Background:**

Spinopelvic dissociation (SPD) is generally caused by high-energy injury mechanisms, and, in the absence of timely diagnosis and treatment, it can lead to chronic pain and progressive deformity. However, SPD is difficult to manage because of its rarity and complexity. In this study, we re-defined SPD according to the mechanism of injuries and biomechanical characteristics of the posterior pelvic ring and developed new classification criteria and treatment principles based on the classification for SPD.

**Methods:**

Between June 2015 and September 2020, 30 patients with SPD which were selected from 138 patients with pelvic fractures were enrolled. Physical examination was performed, classification criteria (301 SPD classification) were developed, and specific treatment standards were established according to the classifications.

**Results:**

The injury mechanisms and co-existing injuries did not significantly differ between the classical SPD patients and expanded SPD patients. The 301 SPD classification criteria covered all the patients. Fixation by biplanar penetration screws was used in 7 patients, 11 patients received fixation by uniplanar penetration screws, 6 patients used sacroiliac compression screws, 3 patients received uniplanar screws combined with sacroiliac compression screws, and open spondylopelvic fixation was used in only 3 patients. According to the Matta criteria, 19, 7, and 4 patients achieved excellent, good, and fair reduction. The Majeed function score of the patients ranged from 9 to 96 points, and the mean score was 72.9 ± 24.6 points.

**Conclusion:**

The expanded definition for SPD is particularly significant for definite diagnosis and prevention of missing diagnosis, based on which the 301SPD classification criteria can more systemically guide the clinical treatment of SPD, increase the treatment efficacy, and reduce surgical trauma.

*Chinese Clinical Trial Registry*: ChiCTR-IPR-16009340.

## Introduction

Spinopelvic dissociation (SPD) includes the discontinuation of the conjunction between the sacrum and bilateral ilium, leading to bi-directional rotational instability between the upper and lower body parts. Severe SPD can manifest as the internal rotation of the spinal axis into the pelvic cavity, which more frequently occurs after high-violence high-energy injuries, also known as a suicidal jumper’s fracture. Roy-Camille et al*.* [1] first reported SPD in 1985 and defined it as an injury secondary to a U-type sacral fracture consisting of the vertical and transverse fractures of the bilateral sacrum. The vertical fracture line mainly affects the sacral foramen, while the transverse fracture line mainly occurs at the S1-2 or S2-3 level, accounting for approximately 2.9% of pelvic fractures [2]. The current understanding of this type of injury is mainly restricted to injuries secondary to complex sacral fractures [3].

In our clinical practice, we encountered many high-violence pelvic fracture patients with discontinuity between the sacrum and bilateral ilium that did not agree with the conventional SPD based on sacral fracture, and some patients’ injuries included the trans-sacroiliac joint and ilium. In addition, several special types of SPD, which have also been reported by other researchers [4], are difficult to be classified and explained as conventional sacral fractures. Also, injury mechanism, biomechanical instability, and concomitant injuries of some cases may be similar to those of the traditional spinal–pelvic separation injury, which could lead to misdiagnosis and a missing diagnosis of non-sacral fracture-based SPD, eventually resulting in severe complications.

The primary aim of surgical treatment [5] for SPD is to reconstruct the structure’s integrity at the posterior pelvic ring. The method used for fixation must be able to resist the complex dislocation tendency in the pelvis, especially the instability from the two kinds of rotation forces. Treatments, including sacroiliac joint screws, penetration screws, and lumbar–sacrum–ilium fixation, are usually selected based on the type of SPD involved [6–9]. Nonetheless, it is of urgent importance to develop optimized and minimally invasive treatments based on the classification of SPD.

In this study, we re-defined SPD according to the mechanism of injuries and biomechanical characteristics of the posterior pelvic ring, which included injuries of the trans-sacroiliac joint and part of transiliac injuries in addition to the transsacral injuries. We also developed new classification criteria and treatment principles based on the classification for SPD. Finally, we also evaluated the accompanied systemic injuries and injuries of peripheral nerves of the pelvis in different types of SPD.

## Materials and methods

### Patients

A total of 138 patients with pelvic fractures were treated in our hospital between June 2015 and September 2020. After preliminary screening by special physical examination and final diagnosis by X-ray and computed tomography (CT), 30 patients with SPD were selected. The inclusion criteria were as follows: (1) ≥ 18 years old; (2) signed informed consent; and (3) with complete dissociation of the unilateral or bilateral ilium and sacrum. The exclusion criteria were: (1) unstable vital signs; (2) severe systemic diseases; (3) coagulation disorders; (4) pregnant women; and (5) unsuitable for the study due to other reasons.

The age, sex, body mass index (BMI), and other general data of the patients were recorded (Table 1). This study was approved by the Ethics Committee of Chinese PLA General Hospital.

**Table 1:** Description of patients’ basic data

Total no. of patients	30
Ages (yr)	40.7 ± 16.3
Sex	
Male	20
Female	10
BMI (kg/m^2^)	23.1 ± 3.3
ISS Score	37.1 ± 13.9
Mechanism	
Fall	12 (40%)
Traffic	9 (30%)
Compression	9 (30%)
Fracture condition	
Single	3 (10%)
Multiple	27 (90%)
Injury condition	
Open	7 (23.3%)
Closed	23 (76.7%)
Temperature (℃)	36.9 ± 0.7
Pulse	92.8 ± 21.4
Mental condition	
Consciousness	27 (90%)
Unconsciousness	3 (10%)
Preoperative DVT	
Yes	0
No	30
Preoperative PE	
Yes	0
No	30
Preoperative resuscitation conditions	
Yes	9 (30%)
No	21 (70%)
Preoperative resuscitation	9 (30%)
The time of preoperative resuscitation (Days)	9.56

### Classification

**Fig. 1:**
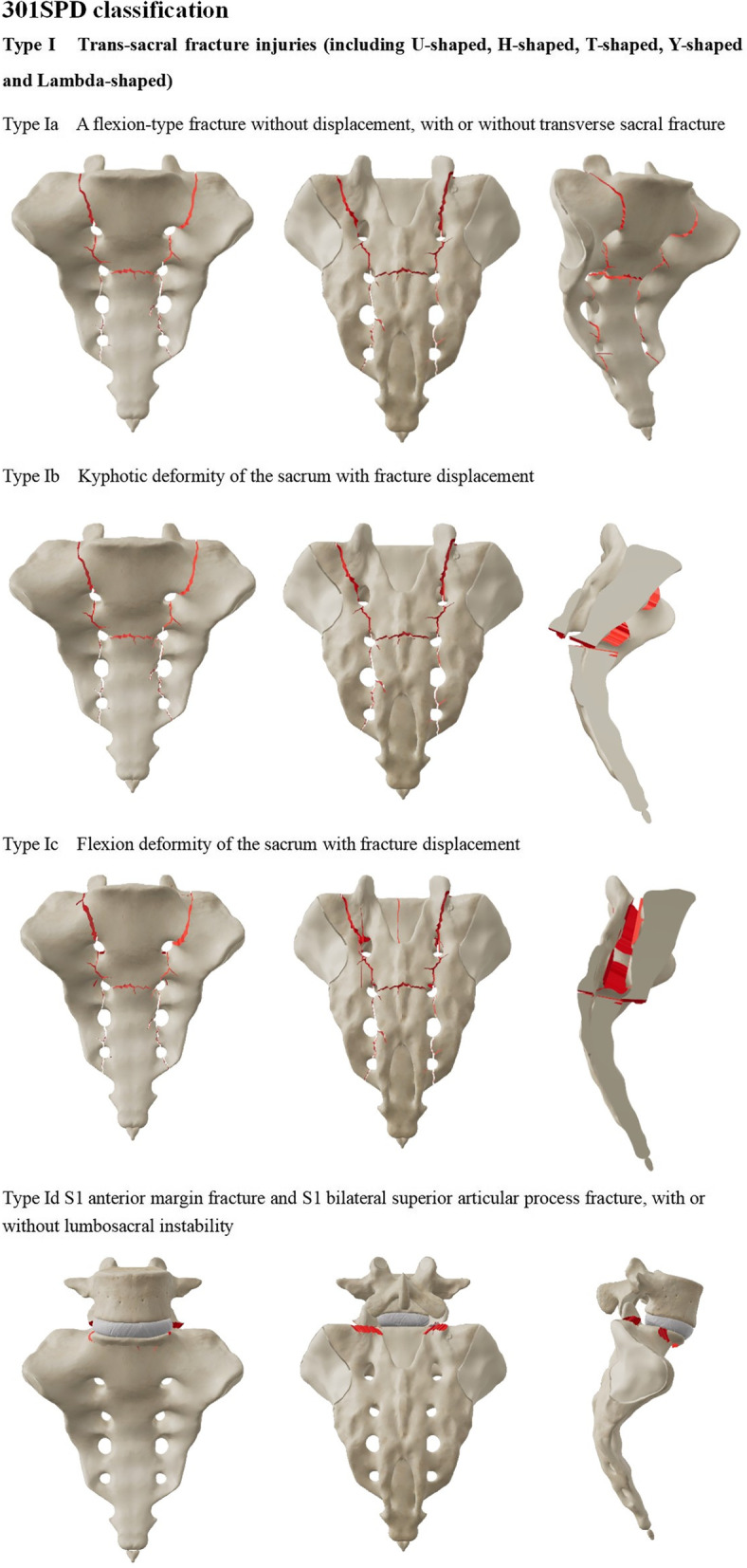

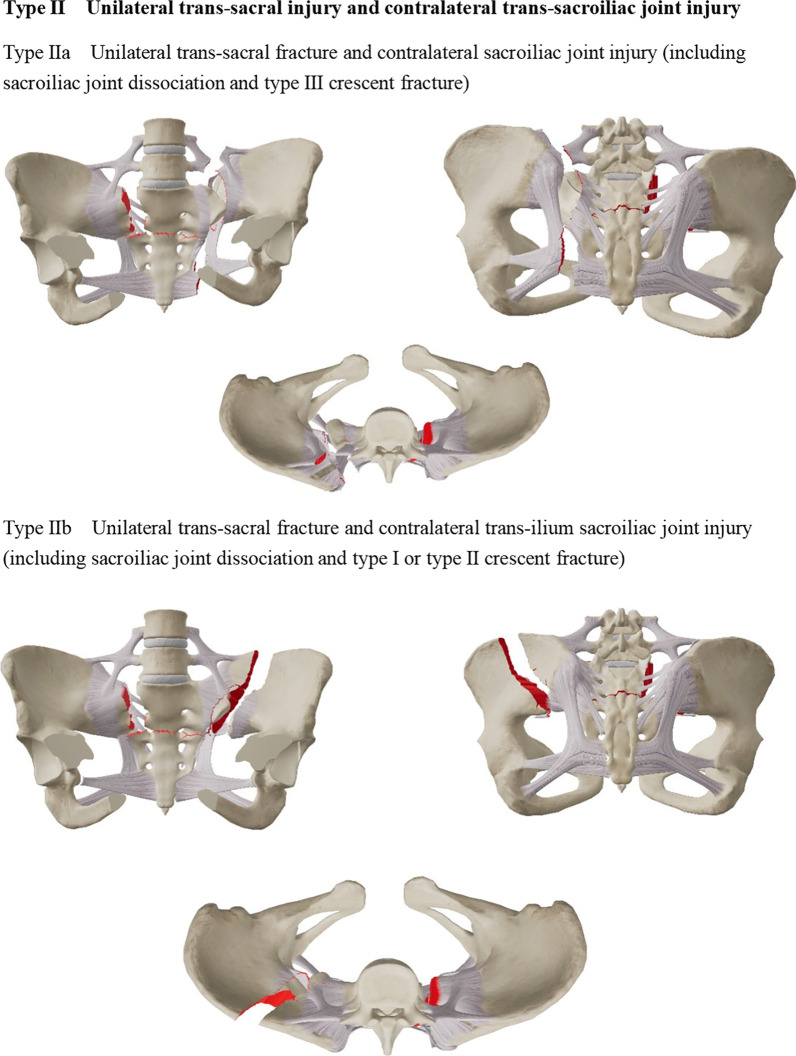

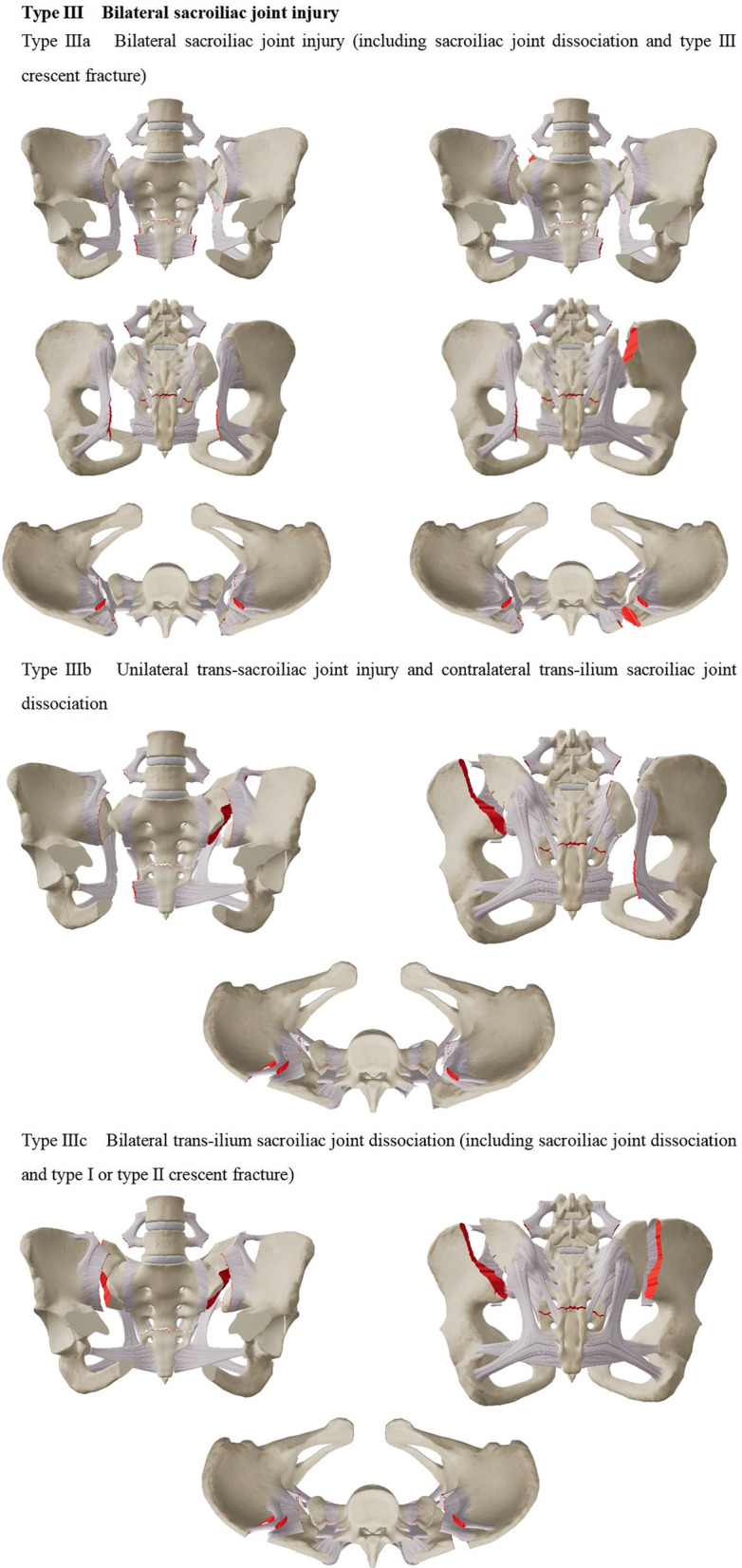
**a–c** The 301 Spinopelvic Dissociation Classification (301SPD classification)

The existing classification is based on a case of sacral fracture with spondylopelvic separation [10, 11]; yet, as not all cases consistent with the mechanism of spinal–pelvic separation injury can be covered, proposing a special classification method for spinal–pelvic separation injuries is necessary. We re-defined the spinal–pelvic separation injury based on the injury of the posterior pelvic ring and biomechanics. In addition to covering the case of transsacral fractures, cases with trans-sacroiliac joint injury and transiliac injury were also included. Typing method, namely 301 Spinopelvic Dissociation Classification (301SPD classification), further analyzed the clinical cases according to the injuries. The 301SPD classification was based on pelvic X-ray and CT imaging data. The types and sub-types of 301SPD classifications are illustrated in detail in Fig. 1a–c.

Compared with the current classification method, the new method covers the cases of transsacral osseous injuries and cases with transsacral iliac joint and transiliac bone injuries. Moreover, the classification is more comprehensive and is based on the mechanism and biomechanics of the posterior pelvic ring injury, which can guide the treatment plan. So to more clearly analyze the effects of different classification criteria in classifying SPD types, we further included the Tile, Young-Burg, and Roy-Camille classifications. The crescent fracture classification was performed for cases in which the fracture line affected the posterior ilium. For cases in which the fracture line involved the sacrum, the Denis zone of the vertical fracture line was performed based on the location of the fracture line regarding the sacral foramina [1, 10–13], and the position of the transverse fracture line was recorded (Table 2).

**Table 2:** Different classifications of the patients

Case	Classification								
	301SPD	Tile	YB classification	Roy-Camille classification	Crescent fracture (Day classification)		Denis classification		Transverse sacral fracture
					R	L	R	L	
1	IIIb	C2	APC-III	N	III	II	N	N	N
2	Ib	C3	CM	II	N	N	II	II	S3
3	Ib	C3	VS	II	N	N	II	II	S2
4	Ia	C3	VS	I	N	N	II	II	S2
5	IIb	C2	LC-III	N	I	N	N	III	S2-3
6	IIIa	C3	CM	N	N	III	N	N	N
7	IIIb	C2	LC-III	N	II	III	N	N	N
8	Ia	C2	CM	N	N	N	II	I	N
9	IIIa	C3	CM	N	III	III	N	N	S3-4
10	IIa	C3	CM	N	N	III	III	N	S2-3
11	Ib	C3	VS	II	N	N	II	II	S2
12	Ic	C3	VS	III	N	N	II	II	S2-3
13	Ia	C2	LC-III	N	N	N	II	II	N
14	IIIc	C3	CM	N	II	I	N	N	N
15	Id	C1	CM	N	III	N	N	N	L5-S1
16	IIIc	C3	CM	N	II	II	N	N	S3
17	IIIa	B1	APC-III	N	N	III	N	N	N
18	Ib	C3	APC-III	II	III	I	N	II	S3
19	IIa	B2	LC-II	N	II	N	I	N	N
20	IIb	C3	LC-III	N	I	N	II	N	N
21	IIb	C2	LC-III	I	N	N	N	II	S4
22	Ib	C3	LC-III	IV	N	N	III	II	S2-3
23	IIIa	B2	LC-II	I	N	N	N	II	S3
24	Id	C3	VS	III	N	N	II	II	S2-3
25	Ic	C2	CM	III	I	N	III	II	S2-3
26	IIb	B3	APC-II	N	I	N	N	N	N
27	IIIc	B2	VS	N	N	III	N	I	N
28	IIb	C2	LC-III	N	N	N	N	II	N
29	Ib	B2	CM	I	N	N	N	III	S4-5
30	Ic	B2	LC-II	III	N	N	II	N	S3

### Physical examination

Physical examination included the pelvic compression and separation test, Faber test of both lower limbs, and drawer test (Table 3).

**Table 3:** The situation of patients’ physical examination

Case	Type				
	Physical examination of the pelvic stability				
	Compression and separation test	Faber test		Drawer test	
1	+	R+	L0	R−	L+
2	+	R−	L0	R−	L+
3	–	R−	L−	R−	L−
4	–	R−	L−	R−	L−
5	+	R+	L−	R+	L−
6	+	R+	L+	R+	L+
7	+	R+	L+	R−	L−
8	+	R+	L−	R+	L−
9	+	R+	L0	R+	L0
10	+	R+	L−	R+	L+
11	–	R−	L−	R−	L−
12	–	R+	L+	R−	L−
13	+	R+	L+	R−	L+
14	+	R0	L+	R0	L+
15	+	R−	L+	R−	L+
16	+	R+	L+	R+	L+
17	+	R+	L++	R+	L++
18	+	R++	L++	R+	L++
19	+	R0	L+	R0	L−
20	+	R+	L+	R+	L+
21	+	R++	L+	R++	L+
22	+	R++	L0	R++	L0
23	+	R+	L0	R+	L0
24	+	R+	L+	R+	L+
25	+	R0	L+	R0	L+
26	–	R+	L0	R+	L0
27	+	R+	L+	R+	L+
28	–	R+	L+	R+	L+
29	+	R0	L0	R0	L0
30	+	R+	L+	R+	L+

### Surgical procedure

General anesthesia was induced. The same team of surgeons performed all the surgeries. After routine disinfection and draping, fracture reduction was performed, and the procedures and methods of the reduction were recorded. The reduction was evaluated by C-arm X-ray imaging, and fixation was performed after the reduction was satisfactory. The fixation method was selected according to the following two principles: (1) to achieve stability between the spine and pelvis; (2) to control the damages and minimize the surgical trauma. According to our previous studies on the biomechanical characteristics of the sacroiliac joint, and following the principle of classification guiding the treatment, the treatment standard was developed according to the 301SPD classification (Table 4).

**Table 4:** The principle of classification guiding the treatment on the basis of force–displacement mechanism

301 SPD classification	Force–displacement	Fixation methods
Type I		
Ia	Non-obvious displacement or kyphotic deformity Relative stability	Transiliac–transsacral penetration screws Trans-sacroiliac screws for fixation of sacroiliac joint
Ib	Non-obvious displacement or Kyphotic deformity Relative stability	Transiliac–transsacral penetration screws Trans-sacroiliac screws for fixation of sacroiliac joint
Ic	Flexion displacement, Lack of support Instability	Posterior spinopelvic fixation (Triangular osteosynthesis)
Id	Lumbosacral displacement	Posterior lumbosacral fixation + Sacral lag screw
Type II		
IIa	Unilateral sacral fracture-displacement, contralateral sacroiliac joint displacement	Fixation of sacroiliac joint (transiliac–transsacral penetration screws and/or sacroiliac screw)
IIb	Unilateral sacral fracture-displacement, contralateral sacroiliac joint displacement accompany ilium fracture	Fixation of sacroiliac joint and/or Repair the ilium
Type III		
IIIa	Bilateral sacroiliac joint displacement	Fixation of sacroiliac joint (transiliac–transsacral penetration screws and/or sacroiliac screw)
IIIb	Unilateral sacroiliac joint displacement, contralateral sacroiliac joint displacement accompany ilium fracture	Fixation of sacroiliac joint and/or Repair the ilium + LC-2 screw
IIIc	Bilateral sacroiliac joint displacement accompany ilium fracture	Fixation of sacroiliac joint and/or Repair the ilium + LC-2 screw

Data on the preoperative time, operation time, blood loss volume, urine volume, intraoperative blood transfusion volume, intraoperative fluid infusion volume, postoperative intensive care unit (ICU) stay, postoperative deep venous thrombosis (DVT), and postoperative pulmonary embolism (PE) were recorded (Table 5).

**Table 5:** Surgical data of intra-operation and post-operation and methods of internal fixation

Variable	Value
Time from injure to surgery (days)	12.0 ± 3.0
Operation time (min)	266.3 ± 120.6
Blood loss (ml)	400.8 ± 488.6
Urine volume (ml)	956.7 ± 70.9
Intraoperative transfusion (u)	2.3 ± 2.5
Colloidal fluid (ml)	755.9 ± 505.0
Crystal fluid (ml)	2286.7 ± 1066.5
Plasma (u)	3.3 ± 2.0
Postoperative ICU duration	14 (46.7)
the time of postoperative ICU duration (days)	5.5 ± 4.9
Postoperative DVT	N
Postoperative PE	N
Internal fixation method	
Anterior pelvic ring	
Infix (Anterior column screw/LC-2 screw)	18 (60.0)
External fixation	1 (3.3)
Double plate for pubic symphysis	4 (13.3)
Posterior pelvic ring	
Penetration screw (S1, S2, S1 + S2)	18 (60.0)
Sacroiliac screw (S1, S2)	6 (20.0)
Penetration screw + sacroiliac screw	3 (10.0)
Spondylopelvic fixation	3 (10.0)

### Follow-up

X-ray and CT images of the pelvis were acquired at the anteroposterior position and inlet and outlet position within 1 week after the surgery. The Matta scores were used to evaluate the degree of dislocation. In addition, the direction of the dislocation of the posterior pelvic ring and the site of injury of the anterior pelvic ring were also recorded [14].

The Majeed pelvis scores [15] at the last follow-up were used to evaluate the quality of life after the treatment (Table 6).

**Table 6:** The Majeed pelvic score at the final follow-up

Case	Follow-up (months)	Majeed score						
		Pain	Work	Sitting	Sexual intercourse	Standing		
						Walking aids	Gait unaided	Walking distance
1	10	30	20	10	N	10	12	6
2	38	30	20	10	4	12	12	12
3	36	25	N	8	N	12	10	12
4	6	30	20	10	N	12	12	12
5	19	30	20	10	4	12	12	12
6	18	30	N	8	N	10	4	8
7	2	N	N	N	N	N	N	N
8	47	5	N	4	N	0	0	0
9	38	25	4	8	N	6	2	8
10	51	25	12	6	N	12	10	12
11	12	25	16	10	4	12	8	12
12	30	30	N	10	N	12	10	12
13	19	30	20	10	4	12	12	12
14	3	25	12	10	N	12	10	10
15	7	30	8	6	N	6	4	4
16	6	25	12	10	3	12	10	8
17	19	30	20	10	3	12	12	12
18	20	30	20	10	4	10	10	6
19	24	30	20	10	N	12	12	12
20	18	30	N	10	N	12	12	10
21	16	30	20	10	4	12	12	8
22	17	30	20	8	N	12	12	10
23	38	25	20	8	N	10	6	4
24	15	25	N	4	N	4	2	4
25	16	25	N	10	4	12	10	8
26	8	25	N	10	4	6	2	4
27	34	30	N	10	4	12	10	10
28	36	30	20	10	4	10	10	8
29	38	25	N	8	N	8	6	4
30	17	30	20	10	N	12	10	10

## Results

### General characteristics

The general characteristics of the patients, including sex, age, BMI, cause of injury, conditions of injury, ISS score, mental conditions, general vital signs, preoperative VDT, preoperative PE, previous resuscitation, and time of the previous resuscitation, are shown in Table 1.

Among 30 patients included in the study, 20 were male and 10 female, with a mean age of 40.7 years (16–68 years). The mean BMI of the patients was 23.1 ± 3.3 kg/m^2^. The causes of the injuries were as follows: fall-induced injuries in 12 patients, traffic injuries in 9 patients, and crush injuries in 9 patients. All the patients had different degrees of a compound fracture. The ISS score was 37.1 ± 13.9 points. The accompanied injuries and fractures of the patients are shown in Table 7. Among patients with SPD, severe injuries were found in 81.3%, and severe lethal injuries were found in 56.3%.

**Table 7:** The associated injuries and fractures of the patients

Variable	Value (%) (n = 30)
Head injury	7 (23.3)
Thoracic injury	19 (63.3)
Abdominal injury	14 (46.7)
Nerve injury	9 (30.0)
Skin injury	11 (36.7)
Upper limb fractures	6 (20.0)
Lower limb fractures	16 (53.3)
Spinal fractures	20 (66.7)

### Classifications of fractures

The 301SPD classifications and other classifications of the patients are shown in Table 2. Among 30 patients with SPD, 14 patients (14/30) were type I SPD, 7 (7/30) were type II SPD, and 9 (9/30) were type III SPD. In addition, there were two patients whose pelvic fracture was type C, but they also had the rupture of bilateral superior articular processes of the S1 vertebra, anterior edge fracture of the S1 vertebra, and sliding dislocation of L5-S1. Robbins et al*.* [4] already reported two cases of such type of SPD, treated as a special type of SPD and classified as the 301SPD-Id type.

### Physical examination

The pelvic stability examination results are shown in Table 3. Compression and separation tests in 24 patients showed (+); 13 patients received bilateral Faber tests, which showed (+); and bilateral drawer tests in 11 patients showed (+).

### Surgical data and efficacy (postoperative X-ray image: dislocation and reduction)

The mean preoperative time of the patients was 12.0 d (4–36 d), and the mean intraoperative blood loss volume was 400.8 ± 488.6 ml (5–2000 ml). The mean intraoperative blood transfusion volume in 18 patients who received blood transfusion was 2.3 ± 2.5U (1.5-10U). The mean operation time was 266.3 ± 120.6 min (85–500 min). Fourteen patients received postoperative ICU treatment, and the mean ICU stay was 5.5 d.

The reduction method and reduction effects are shown in Table 8. Seven patients had an initial dislocation > 15 mm, and twenty-three patients had an initial dislocation < 15 mm. After the surgical reduction, the evaluation by Matta reduction criteria showed that the reduction was excellent, good, and fair in 19, 7, and 4 patients, respectively, and the good reduction rate was 86.7%.

**Table 8:** The reduction method and Matta score of patients before and after operation

Variable	Value (%) (n = 30)
Reduction method	
Open	9 (30)
Close	21 (70)
Tornetta–Matta score	
Excellent	19 (63.3)
Good	7 (23.4)
Fair	4 (13.3)

**Fig. 2:**
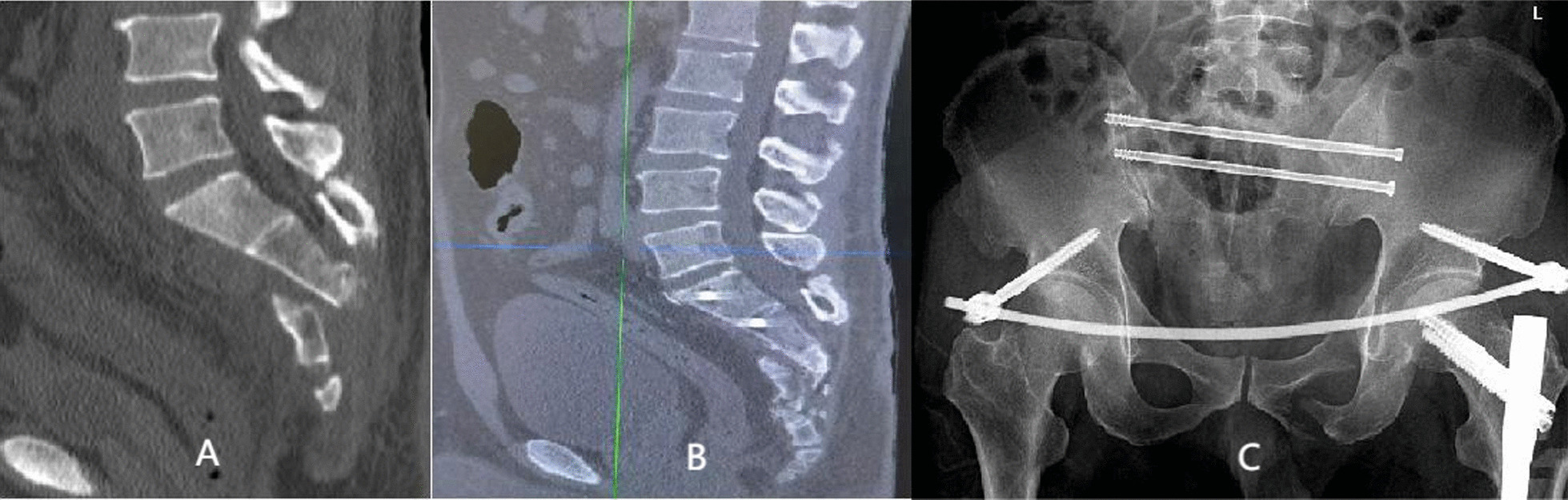
Case 2. A 44-year-old man who was injured from a fall from a height underwent S1 and S2 transiliac–transsacral screw fixation for a 301SPD type Ib fracture. **A** Preoperative CT scan of the sacrum in the midline sagittal plane shows kyphotic deformity. **B** Postoperative CT scan midline sagittal image shows unreduction of fracture. **C** The radiological examination image shows that the fracture was fixed satisfactorily

**Fig. 3:**
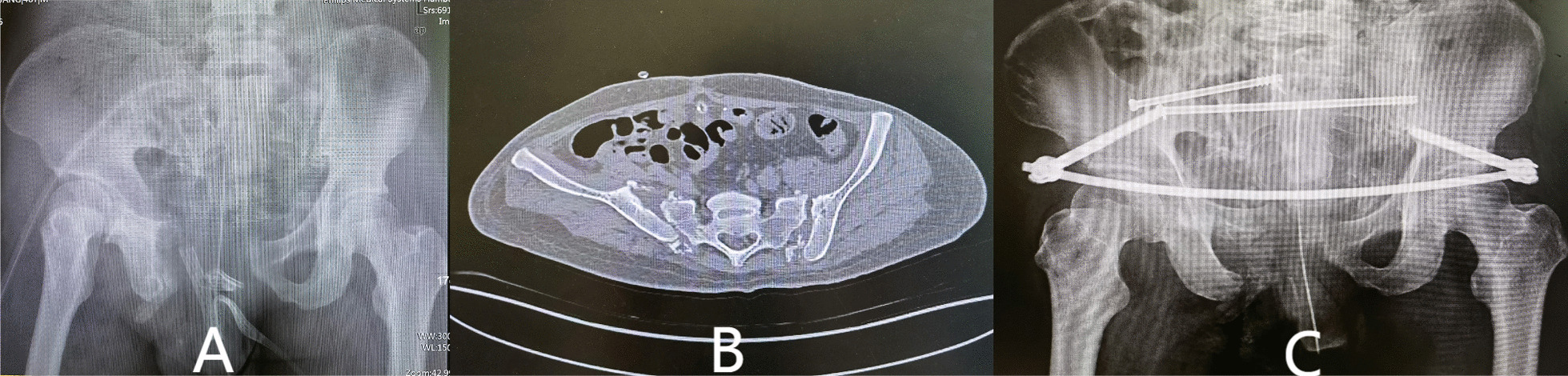
Case 6. A 46-year-old man who was injured from compression underwent S1 sacroiliac screw and S2 transiliac–transsacral screw fixation for a 301SPD type IIIa fracture. **A** Preoperative X-ray shows bilateral sacroiliac joint injury. **B** Preoperative CT scan cross-sectional image shows displacement of fracture. **C** The X-ray image shows that the fracture was fixed satisfactorily

**Fig. 4:**
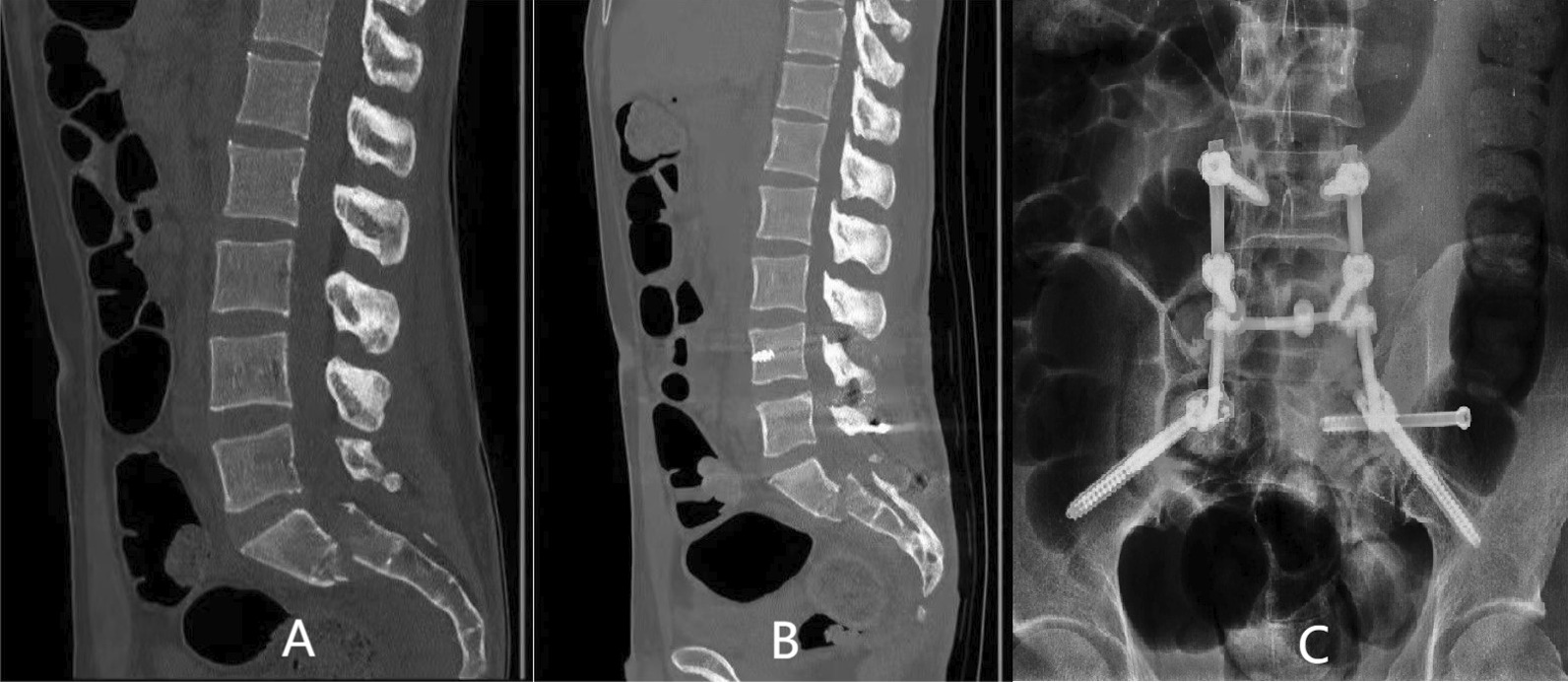
Case 12. A 16-year-old man who was suffered from fall injury from height underwent spinopelvic fixation and S1 sacroiliac screw fixation for a 301SPD type Ic fracture. **A** Preoperative CT scan of the sacrum in the midline sagittal plane shows anterior flexion displacement. **B** Postoperative CT scan midline sagittal image demonstrates good reduction of fracture. **C** The radiological examination image shows that the fracture was reduced and fixed satisfactorily

**Fig. 5:**
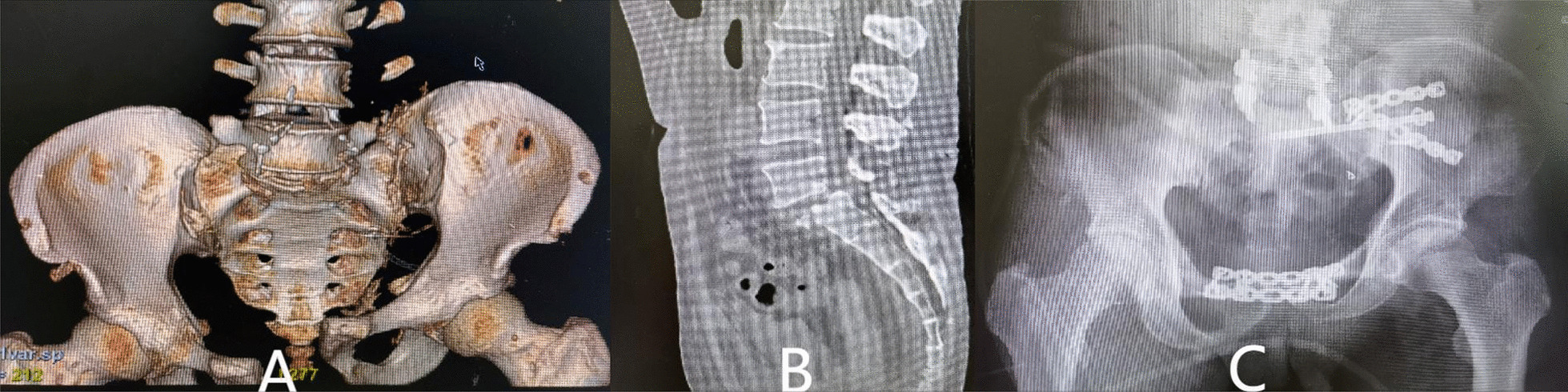
Case 15. A 52-year-old man who was suffered from compression injury underwent lumbosacral fixation and S1 sacroiliac screw fixation for a 301SPD type IIIa fracture. **A** Preoperative X-ray shows bilateral sacroiliac joint injury. **B** Preoperative CT scan cross-sectional image shows displacement of fracture. **C** The X-ray image shows that the fracture was fixed satisfactorily

**Fig. 6:**
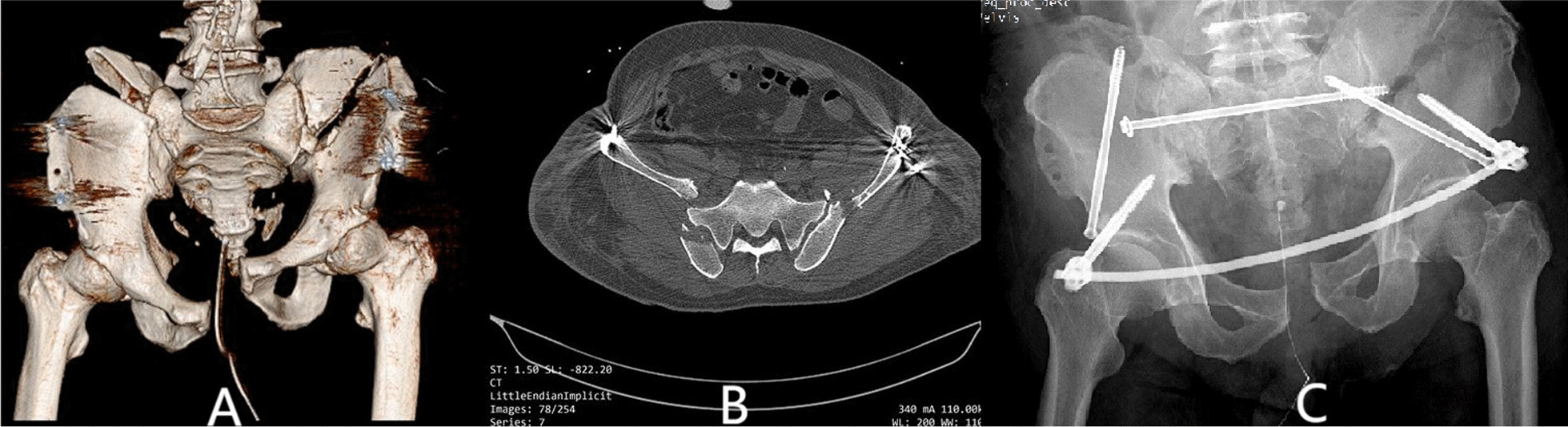
Case 14. A 60-year-old man who was injured from compression underwent S1 transiliac–transsacral screw combined with LC-2 screw fixation for a 301SPD type IIIc fracture. **A** Preoperative 3d-CT scan shows bilateral sacroiliac joint displacement accompany ilium fracture-displacement. **B** Preoperative CT scan cross-sectional image shows type II crescent fracture on the right and type I crescent fracture on the left. **C** The radiological examination image shows that the fracture was reduced and fixed satisfactorily

Seven patients received fixation of posterior pelvic rings, and 23 received combined fixation of anterior and posterior pelvic rings. The methods for fixation of the anterior ring included infix fixation (n = 18), external fixation (n = 1), and plate fixation (n = 4). The methods for fixation of the posterior pelvic ring included fixation by biplanar penetration screws (Fig. 2), uniplanar screws combined with sacroiliac compression screws (Fig. 3), open spondylopelvic fixation (Fig. 4), lumbosacral fixation (Fig. 5), and penetration screw combined with LC-2 screw (Fig. 6). Fixation by uniplanar transsacral–transiliac penetration screws was in 11 patients (7 patients with S1 and 4 with S2), S1 + S2 biplanar transsacral–transiliac penetration screws in 7 patients, sacroiliac compression screws in 6 patients, transsacral–transiliac penetration screws combined with sacroiliac compression screws in 3 patients, and open spondylopelvic fixation in 3 patients (Table 5).

### Evaluation of quality of life

The 30 patients were followed for 2–51 months, and the mean follow-up time was 21.9 months. No complications such as wound hematoma, wound infection, posterior iliac pressure sore, urinary infection, rupture of connecting rod or screw, and second dislocation of fracture were found. The sacroiliac screws were not removed in all patients. Six patients reported discomfort in the fixation area but did not receive specific treatment. The consequent follow-up alleviated all the symptoms.

All 30 patients completed the Majeed pelvis scores evaluation at the last follow-up (Table 6). The Majeed scores of the patients ranged from 9 to 96 points, and the mean score was 72.93 points. The Majeed score was not available for 1 patient (the patient died), 1 patient was with no postoperative movement (T12 fracture accompanied with paraplegia), and 6 patients were with a slight movement disorder. All the other patients could exercise, their recoveries were satisfactory, and they could normally engage in daily living activities. All the patients (except for the dead patient and the patient with paraplegia) were satisfied with surgery. Three patients reported that their mental status significantly improved after the surgery.

## Discussion

We have supplemented and improved the definition, classification, and targeted treatment of spinal–pelvic separation injuries through research. We found that in some cases injury mechanisms, biomechanical instability, and concomitant injuries were similar to those of the traditional spinal–pelvic separation injury, which led to clinical concerns. Insufficient understanding of the severity leads to missed diagnosis, which in turn affects the follow-up treatment effect and can lead to serious complications. Our data provide a more in-depth understanding of spinal–pelvic separation injury and expand the definition of spinal–pelvic separation injury, which is of great significance for a clear diagnosis. On this basis, applying a new classification method (301 SPD) can guide clinical treatment and improve treatment effects.

Complex SPD, generally caused by high-energy injury mechanisms [16, 17], is associated with a high injury rate and high mortality rate [18–20]. The clinical manifestations include severe systemic injuries, which are generally accompanied by craniocerebral injury, injuries of organs in thoracic, abdominal, and pelvic cavities, and coexistent fractures of limbs and spine. Previous studies [20] reported organ injuries in 42% of SPD patients, thoracic injuries in 37% of them, closed craniocerebral injuries in 21%, musculoskeletal injuries (including limbs) in 63%, an anterior pelvic fracture in 52%, other vertebral fractures in 47%, and spinal injuries in 16% patients. Due to many complex injuries, fatal injuries were first examined, which can easily lead to a missed diagnosis of spinal and pelvic separation injuries. Once the SPD injury is suspected, the corresponding imaging examination should immediately be marked to further confirm the SPD injury diagnosis. The evaluation of the conditions of soft tissues is also extremely important for patients with complex, high-energy lumbosacral injuries [21].

The comorbidities and co-existent injuries of the included patients were further analyzed; 7 (7/30) patients were with craniocerebral injury and disturbance of consciousness, 19 (19/30) patients were with rib fractures on both sides, hemopneumothorax, and pulmonary contusion, 11 (11/30) patients were with skin and soft tissue injuries (including degloving skin injury in 2 patient and open wound in 9 patients). In addition, all 30 patients also had fractures of other body parts, which is consistent with the results reported by previous studies [19–21]. These findings demonstrated that the new SPD diagnostic definition was highly consistent with the conventional classification from the aspect of accompanying injuries.

SPD is caused by the axial vertical stress and shearing force of the spine relative to the bilateral ilium. The description of SPD classification in previous studies generally focused on the complex fractures of the sacrum [22–26], but did not include the conditions of transsacral injury on the one side and trans-sacroiliac joint injury on the other side of the conditions of trans-sacroiliac joint injuries on bilateral sides. Our results showed that out of the 30 patients, 7 were with transsacral injury on the one side and trans-sacroiliac joint injury on the other side, thus accounting for 23.3% of all included cases, while nine patients were with trans-sacroiliac joint injuries on bilateral sides, accounting for 30%. As the soft tissue healing of sacroiliac dissociation and ligament complex is more difficult to be predicted than bone healing, these two types of SPD should also be given adequate attention.

The severity of SPD is directly associated with neurological risks, surgical hemorrhage, and postoperative complications [27]. In addition, palpation of the sacrum–pelvis area, anogenital examination (to rule out hidden open fracture or dystonia of sphincter of the anus), signs of colon and bladder dysfunction, and even cauda equina syndrome [28] are also indirect signs suggestive of SPD in clinical practice.

The new 301SPD classification method was developed based on the anatomic and biomechanical characteristics and the mechanisms of injuries. According to the new 301SPD classification criteria, we specifically developed different fixation methods for different types of SPD. The core concept was to control the shearing and rotation disability of the sacrum. Our previous study [29] suggested the following: (1) If the fracture type has no obvious displacement or kyphosis displacement, and the fracture is accompanied by relatively stable support (such as Type Ia or Ib), double penetrating screw fixation should be recommended; (2) if there is unilateral sacroiliac joint separation (such as type II or III), penetrating screw combined with sacroiliac lag screw fixation should be used; (3) if the fracture type is anterior flexion displacement (such as Type Ic) and the fracture is unstable because of lack of support, regardless of reduction or not, spondylopelvic fixation should be recommended; and (4) if lumbosacral displacement occurs (such as Type Id), lumbosacral fusion fixation should be used (Table 4). The transsacral–transiliac penetration screws [6] offer a long arm of force for fixation; they can disperse the load and reduce the stress of the tip of screws, thus decreasing the risk of dislocation, and can resist the rotation and shearing stress [30–33]. Sacroiliac compression screws are commonly used for injuries of SPD [34], and open lumbosacral fixation was used for patients with lumbosacral instability [35, 36]. The corresponding fixation according to the SPD types achieved good clinical efficacy in the postoperative evaluation and clinical follow-up (mean follow-up time: 21.9 months).

The major limitations of this study are following: (1) low sample size; (2) the follow-up time was not the same for all patients. Thus, a prospective controlled experiment with a larger sample and longer follow-up should be performed to evaluate the observer consistency of the classification method and to further verify the effect of the treatment of patients with spinal–pelvic separation under the guidance of our research.

## Conclusion

The expanded definition of SPD is significant for establishing a definite diagnosis and preventing missing diagnoses. 301SPD classification criteria can be used to more systemically guide the clinical treatment of SPD, increase the treatment efficacy, and reduce surgical trauma.

## Data Availability

The data supporting this study’s findings are available from the Chinese PLA General Hospital, but restrictions apply to the availability of these data, which were used under license for the current study, and so are not publicly available. Data are, however, available from the authors upon reasonable request and with permission of the above hospital, China.

## Abbreviations

SPD: Spinopelvic dissociation
BMI: Body mass index
ICU: Intensive care unit
DVT: Deep venous thrombosis
PE: Pulmonary embolism
CT: Computed tomography
